# A comparison of Mini-FLOTAC and McMaster techniques, overdispersion and prevalence of parasites in naturally infected North American bison (*Bison bison*) in the USA

**DOI:** 10.1016/j.crpvbd.2022.100103

**Published:** 2022-11-08

**Authors:** William L. Johnson, Samantha Reynolds, Colton L. Adkins, Bradly Wehus-Tow, Jameson Brennan, Catherine B. Krus, Danielle Buttke, Jeff M. Martin, Jeba R.J. Jesudoss Chelladurai

**Affiliations:** a Diagnostic Medicine/Pathobiology, Kansas State University College of Veterinary Medicine, Manhattan, KS, USA; b Department of Animal Science, South Dakota State University, Rapid City, SD, USA; c Department of Clinical Sciences, Colorado State University, Fort Collins, CO, USA; d National Parks Service, Fort Collins, CO, USA; e Center of Excellence for Bison Studies, South Dakota State University, Rapid City, SD, USA

**Keywords:** Bison, Fecal test, McMaster, Mini-FLOTAC, Overdispersion

## Abstract

Several quantitative diagnostic techniques are available to estimate gastrointestinal parasite counts in the feces of ruminants. Comparing egg and oocyst magnitudes in naturally infected samples has been a recommended approach to rank fecal techniques. In this study, we compared the Mini-FLOTAC (sensitivity of 5 eggs per gram (EPG)/oocysts per gram (OPG)) and different averaged replicates of the modified McMaster techniques (sensitivity of 33.33 EPG/OPG) in 387 fecal samples from 10 herds of naturally infected North American bison in the Central Great Plains region of the USA. Both techniques were performed with fecal slurries homogenized in a fill-FLOTAC device. In the study population, prevalence of strongyle eggs, *Eimeria* spp. oocysts, *Moniezia* spp. eggs and *Trichuris* spp. eggs was 81.4%, 73.9%, 7.5%, and 3.1%, respectively. Counts of strongyle eggs and *Eimeria* spp. oocysts obtained from 1 to 3 averaged technical replicates of the modified McMaster technique were compared to a single replicate of the Mini-FLOTAC. Correlation between the two techniques increased with an increase in the number of averaged technical replicates of the modified McMaster technique used to calculate EGP/OPG. The correlation for *Moniezia* spp. EPG when averaged triplicates of the modified McMaster technique were compared to a single replicate of the Mini-FLOTAC count was high; however, the correlation for *Trichuris* spp. eggs was low. Additionally, we used averaged counts from both techniques to show the overdispersion of parasites in bison herds.

## Highlights

• Mini-FLOTAC is an acceptable alternative to the McMaster for bison parasites.
• Increasing technical replicates of McMaster increases correlation with mini-FLOTAC.
• Gastrointestinal parasites are overdispersed in bison.

## Graphical abstract

**Figure:**
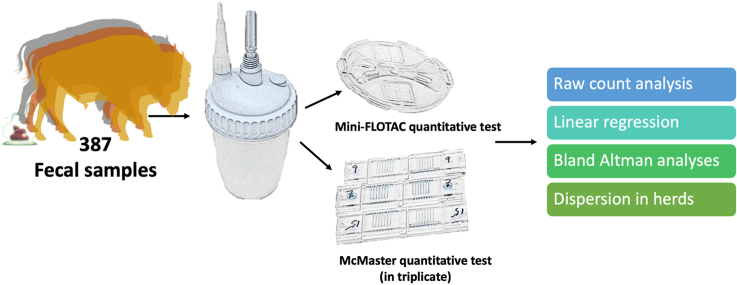

## 1 Introduction

The North American bison (*Bison bison bison*) is an understudied ungulate species that has historically been an important part of the ecosystem of the USA. Due to human activities, bison numbers reduced in the late 1880s to only a few hundred animals (Hornaday, 1889). However, there has been a recent push to re-establish herds in their historic habitat for ecological, economic, and cultural reasons (Shamon et al., 2022). Re-established herds face many challenges including those posed by infectious agents that are shared with sympatric cattle (Tessaro, 1989; Van Vuren & Scott, 1995).

Gastrointestinal parasites affect North American bison and may cause clinical disease (Eljaki et al., 2016). It is estimated that 5% of all bison deaths can be attributed to parasitic infections (USDA-APHIS, 2016). Since the 1990s, there has been limited parasitology research in farmed and free-roaming bison in the USA (Eljaki et al., 2016; Wiese et al., 2021), while several studies describe bison parasites in Canadian herds (Woodbury & Lewis, 2011; Woodbury et al., 2012, 2014; Avramenko et al., 2018). As bison become more common due to ongoing restoration efforts, applying modern parasitological techniques to this host species could provide useful insights for North American bison veterinarians and bison producers.

Diagnosis of parasitism with gastrointestinal nematodes and protozoans in ruminants is important for evaluating individual and herd health and can be a part of good management practice (Verocai et al., 2020). Quantitative fecal techniques are preferred over qualitative techniques to evaluate the level of infection in herd animals. Fecal egg and oocyst counts in grazing animal herds can be used to estimate production and economic losses, to understand stocking densities, to make management decisions, such as the requirement for anthelmintic treatments, and to evaluate anthelmintic efficacy and parasite resistance using fecal egg count reduction tests (Nielsen, 2021).

Routinely used quantitative tests in ruminants include the McMaster, Mini-FLOTAC, Stoll’s, and Wisconsin double centrifugation techniques and several modifications to the protocols originally described (Paras et al., 2018). Recently, the McMaster technique has been compared to the Willis technique in fecal samples of European bison (*Bison bonasus*) (Gałązka et al., 2022). However, only the Wisconsin, modified Wisconsin centrifugation, and the McMaster fecal techniques have been used to quantify parasites in North American bison fecal samples (Dies & Coupland, 2001; Eljaki et al., 2016; Avramenko et al., 2018; Wiese et al., 2021). The performance of the Mini-FLOTAC technique has not been tested with bison fecal samples.

The McMaster technique is an older technique which may use a double-chambered slide allowing for a total volume of 0.3 ​ml to be examined (Gordon & Whitlock, 1939). Commonly used diagnostic sensitivities of the McMaster include 25 eggs per gram/oocysts per gram (EPG/OPG; hereafter referred to as ‘EPG’) and 50 EPG (Rinaldi et al., 2014; Bortoluzzi et al., 2018), but can be modified to 10, 15, 33.3 EPG (Levecke et al., 2011). The Mini-FLOTAC technique is a newer technique that utilizes a double-chambered disc allowing for a volume of 2 ​ml to be examined, with an analytical sensitivity of 5 EPG (Cringoli et al., 2017). The Mini-FLOTAC, when used with the recommended homogenizer device called the fill-FLOTAC, has been reported to have higher accuracy, precision and egg recovery compared to the McMaster technique (Noel et al., 2017; Paras et al., 2018).

It is important to note that analytical sensitivities and specificities of quantitative fecal techniques can be modified by changing the ratios of fecal sample to floatation solution (Vadlejch et al., 2011). Additionally, choice of flotation solution, consistency, and volume of feces analyzed can alter the reliability of estimating fecal egg counts (Cringoli et al., 2004), resulting in variations in accuracy (Nápravníková et al., 2019). This may have important implications on the interpretation of the results and downstream decision making. Fecal egg counts can be used as biomarkers of parasite infection in field studies to determine anthelmintic efficacy (Geurden et al., 2022). The World Association for the Advancement of Veterinary Parasitology (WAAVP) guidelines suggest that test selection for field studies be based on a minimum total number of eggs counted on the slide/chamber to increase the diagnostic power of fecal egg count reduction determination (Geurden et al., 2022). For ruminants, the recommended number is 200 eggs, although 50 eggs is deemed the absolute minimum (Kaplan, 2020). If this minimum number cannot be counted in a single replicate of the test, additional slides or chambers must be counted and averaged, until the pre-set raw egg count threshold is exceeded (Kaplan, 2020; Geurden et al., 2022).

While there is high correlation between fecal eggs counts, worm burdens, and parasitic effects in infections with pathogenic *Haemonchus contortus* (Le Jambre, 1995), the correlation is lower with other nematodes such as *Cooperia* spp. and may vary with season (González-Garduño et al., 2013). These factors in addition to variation of parasite burdens within a herd, referred to as overdispersion, necessitate careful interpretation of fecal egg counts. Overdispersed parasite burdens have direct effects on transmission dynamics (Churcher et al., 2005). Overdispersion of strongyles in bison have been shown in one herd (Wiese et al., 2021). However, there is a lack of knowledge about the distribution of strongyles in many herds and of other parasites such as *Eimeria* spp., *Moniezia* spp., and *Trichuris* spp. in bison.

The aim of this study was to compare the performance of two quantitative fecal techniques, the Mini-FLOTAC and averaged technical replicates (1–3 replicates) of the modified McMaster, to quantify strongyle eggs, *Eimeria* spp. oocysts, *Moniezia* spp. eggs, and *Trichuris* spp. eggs present in bison fecal samples. Additionally, dispersion of strongyle eggs, *Eimeria* spp. oocysts, *Moniezia* spp. eggs, and *Trichuris* spp. eggs in the herds was analyzed.

## 2 Materials and methods

### 2.1 Sample collection

Fecal samples from a total of 387 farmed or free-roaming North American bison, from 10 herds in 7 states (Iowa, Illinois, Indiana, Kansas, Missouri, Oklahoma and South Dakota) were analyzed in this study. Samples were collected per-rectally or from freshly voided fecal material within 10 ​min of deposition. Samples were collected between August 2021 and January 2022. Samples were double-bagged in plastic resealable bags and shipped to the laboratory. Upon arrival, the samples were placed in a 4 ​°C refrigerator in the laboratory until quantitative analysis was performed.

### 2.2 Quantitative techniques

Quantitative fecal floats were performed on the 387 samples using Mini-FLOTAC and modified McMaster techniques. For each sample, 5 ​g of feces were combined with 45 ​ml of Sheatherʼs solution (specific gravity of 1.275) and homogenized in a fill-FLOTAC as described in the Mini-FLOTAC protocol (Cringoli et al., 2017). For the Mini-FLOTAC technique, 1 ​ml ​× ​2 of the fecal slurry from the fill-FLOTAC was filled into the 2 flotation chambers of the Mini-FLOTAC disc, to obtain a sensitivity of 5 EPG. For the modified McMaster technique, 0.3 ​ml ​× ​2 of the same fecal slurry was filled into a standard 2-chamber McMaster slide, to obtain a sensitivity of 33.33 EPG. For each sample, the McMaster techniques were run in triplicate from the same fecal slurry. All parasite eggs/oocysts under the grid were counted in the McMaster and the Mini-FLOTAC chambers at 10× ​magnification using an Olympus CX31 (Olympus, Japan) and/or a Zeiss Axiostar plus (Carl Zeiss AG, Germany) microscope.

### 2.3 Statistical analysis

Raw counts were summarized in Microsoft Excel and data were found to be positively skewed. The ‘N-1’ Chi-square test to compare sample proportions on zero raw counts between the two techniques was performed in MedCalc version 20.116 (MedCalc Software Ltd, 2022). Linear regression and correlation (Pearson) analyses were performed to compare the Mini-FLOTAC counts, and average McMaster counts at different replicate levels using R packages *ggplot2*, *ggprism*, *ggmisc*, and *ggpubr*. Technical triplicates were averaged across the three McMaster counts and compared against the single Mini-FLOTAC value. Technical duplicates were averaged in combination across the three replicates (replicate 1 and 2, replicate 2 and 3, and replicate 1 and 3) and compared to the single Mini-FLOTAC value. Single technical replicates of McMaster were compared to the single Mini-FLOTAC value. Linear regression coefficients were compared using an analysis of covariance (ANCOVA) in JASP v0.16.2 (JASP Team, 2022). Pearsonʼs correlation coefficient values were compared using cocor version 1.1–3 (Diedenhofen & Musch, 2015). Bland-Altman analyses were performed to compare the different technical replicates (1 to 3) of McMaster counts to the Mini-FLOTAC using the R package using *blandr* and graphs were produced using R packages *ggplot2* and *ggprism*. Bland-Altman plots compare the two techniques by plotting the average count (mean) for each sample against the differences between them, with the upper and lower limits of agreement indicating the range within which 95% of differences of the second method fall compared to the first.

Dispersion of strongyle nematodes, *Eimeria* spp., *Moniezia* spp. and *Trichuris* spp. within each herd was analyzed with ridge plots made using the R packages *ggplot2*, *ggridges*, and *ggprism*. Prevalence was calculated in Microsoft Excel. Skewness was calculated with JASP v0.16.2 (JASP Team, 2022).

## 3 Results

### 3.1 Comparison of raw strongyle egg counts

A detailed analysis of raw strongyle egg counts was performed because test selection for strongyle egg counts is a part of the WAAVP guidelines. Of the 387 samples analyzed using the two techniques, raw egg counts recovered by the single replicate of Mini-FLOTAC were higher than the sum of three McMaster counts in 352 (91.0%) samples. Zero egg counts were recovered in 75 out of 387 (19.4%) total Mini-FLOTAC discs read and in 501 out of 1161 (43.2%) McMaster slides read. Zero egg counts reduced to 102 out of 387 (26.4%) when the three technical replicates of McMaster were averaged. Zero egg counts with a single replicate of the Mini-FLOTAC (19.4%) were significantly lower than zero egg counts with the single replicate of McMaster (43.2%) (“N-1” Chi-square test of proportions; *χ*^2^ ​= ​70.2, *P* ​< ​0.0001) but zero oocyst counts were not significantly different when compared to the zero counts obtained in averaged triple replicates of McMaster (26.36%) (“N-1” Chi-square test of proportions; *χ*^2^ ​= ​5.354, *P* ​= ​0.0207).

Raw egg counts recovered in a single replicate of the Mini-FLOTAC were higher in 349 out of the 387 samples (90.2%) than the sum of the three replicates of McMaster test. Raw strongyle egg counts with the single replicate of the Mini-FLOTAC prior to application of the multiplication factor exceeded the minimum threshold of 50 EPG in 24 out of the 387 samples (6.2%). Raw strongyle egg counts with the sum of the three replicates of McMaster test exceeded the threshold of 50 EPG in 8 out of 387 samples (2.1%).

### 3.2 Comparison of raw *Eimeria* spp. oocyst counts

Of the 397 samples analyzed using the two techniques, zero oocysts were recovered in 291 out of 387 (75.2%) total Mini-FLTOAC discs read and in 999 out of 1161 (86%) McMaster slides read. Zero oocyst counts reduced to 306 out of 387 (79.1%) when the three technical replicates of McMaster were averaged. Zero oocyst counts with a single replicate of the Mini-FLOTAC (75.2%) were significantly lower than the single replicate of McMaster (86%) (“N-1” Chi-square test of proportions; *χ*^2^ ​= ​24.321, *P* ​< ​0.0001), but zero oocyst counts were not significantly different when the averaged triple replicates of McMaster (79.1%) was compared (“N-1” Chi-square test of proportions; *χ*^2^ ​= ​1.581, *P* ​= ​0.209). Raw counts recovered in a single replicate of the Mini-FLOTAC were higher in 352 out of 387 samples (91%) than the sum of the three replicates of McMaster test.

### 3.3 Comparison of Mini-FLOTAC and McMaster for strongyle eggs

**Fig. 1:**
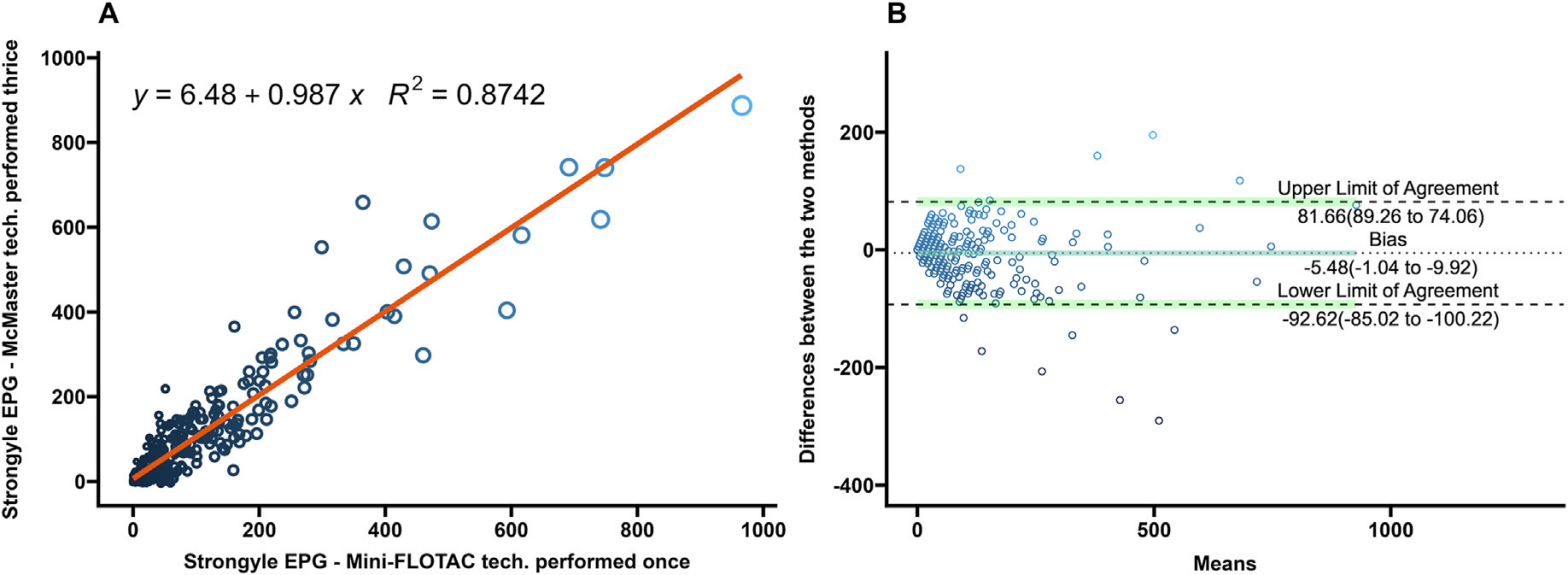
**A** Scatterplot and linear regression for strongyle-type eggs determined by Mini-FLOTAC and three averaged technical replicates of McMaster techniques. Equations and regression coefficients are included. **B** Bland-Altman plot comparing the differences of the strongyle-type egg counts of the Mini-FLOTAC and three averaged technical replicates of McMaster techniques. Upper limit of agreement, lower limit of agreement, and bias are included.

**Fig. 2:**
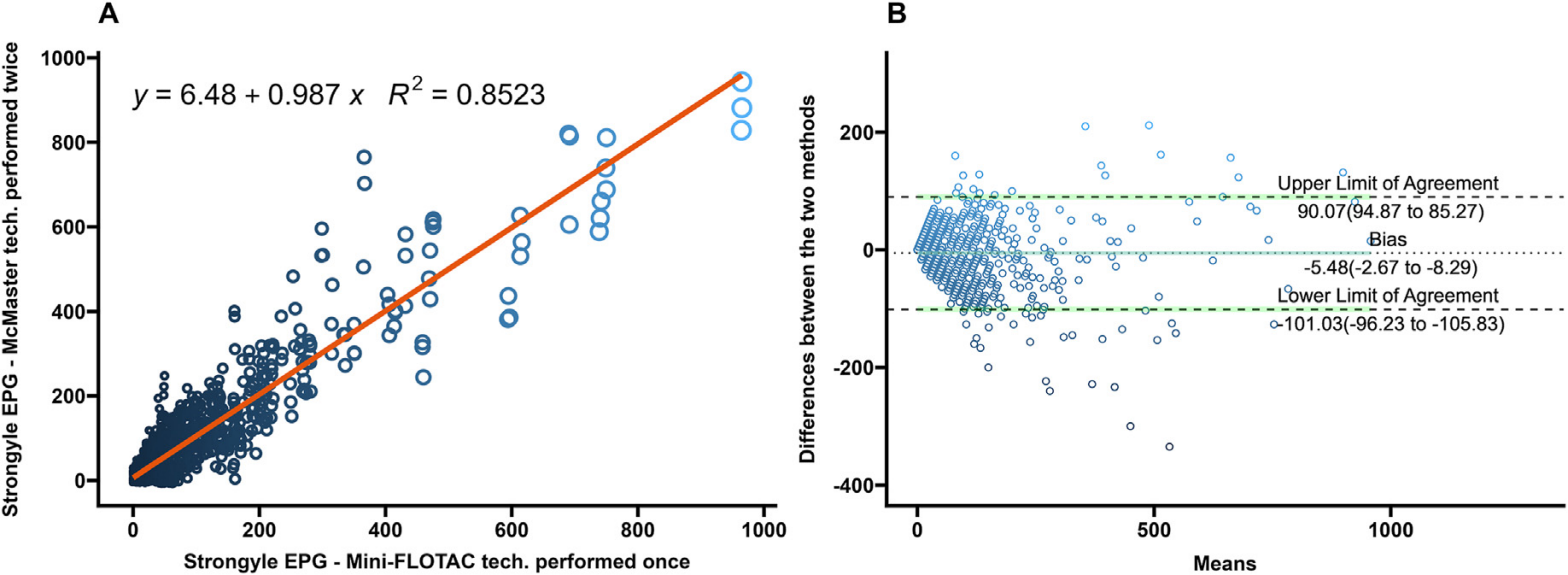
**A** Scatterplot and linear regression for strongyle-type eggs determined by Mini-FLOTAC and two technical replicates of McMaster techniques. Equations and regression coefficients are included. **B** Bland-Altman plot comparing the differences of the strongyle-type egg counts of the Mini-FLOTAC and two technical replicates of McMaster techniques. Upper limit of agreement, lower limit of agreement, and bias are included.

**Fig. 3:**
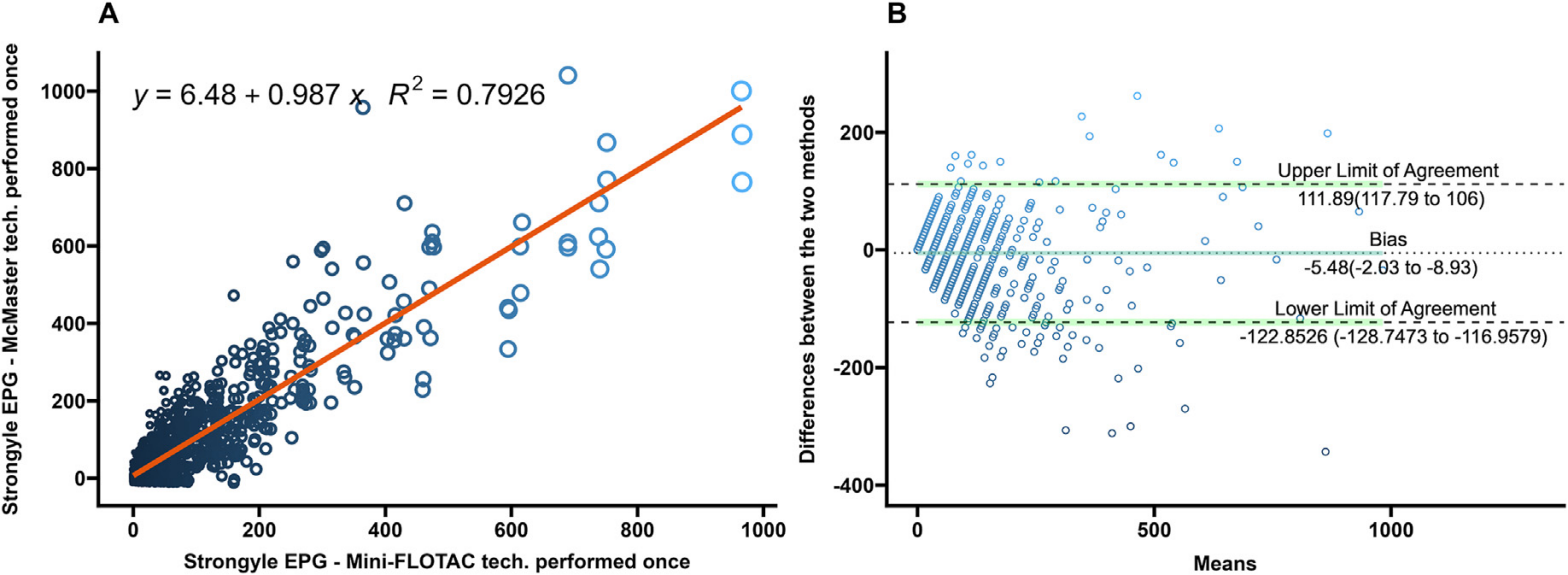
**A** Scatterplot and linear regression for strongyle-type eggs determined by Mini-FLOTAC and one technical replicate of McMaster technique. Equations and regression coefficients are included. **B** Bland-Altman plot comparing the differences of the strongyle-type egg counts of the Mini-FLOTAC and one technical replicate of McMaster technique. Upper limit of agreement, lower limit of agreement, and bias are included.

A comparison of the performance of the Mini-FLOTAC and different replicates of the McMaster techniques on strongyle eggs from bison fecal samples performed after the multiplication factor is summarized in Table 1. The highest agreement between the two techniques occurred when the Mini-FLOTAC was compared to the average of technical triplicates of the McMaster technique (*R*^2^ ​= ​0.87; Pearsonʼs *r* ​= ​0.94) (Fig. 1A). Agreement was similar when an average of technical duplicates was compared (*R*^2^ ​= ​0.85; Pearsonʼs *r* ​= ​0.92) (Fig. 2A) and lowest when a single technical replicate of McMaster was compared (*R*^2^ ​= ​0.79; *r* ​= ​0.89) (Fig. 3A).

**Table 1:** Summary of statistical comparisons between Mini-FLOTAC and McMaster techniques from this study

MiniFLOTAC	McMaster	Correlation coefficient *r*	Regression coefficient *R*^2^	Average difference in EPG (95% CI)	Non-parametric limit of agreement (95% CI)
Strongyle eggs					
Once	Thrice	0.935 (0.921–0.946)	0.8739	−5.480 (−9.924; 1.036)	Upper: 81.664 (74.063; 89.264);
					Lower: −92.624 (−100.225; −85.023)
Once	Twice	0.923 (0.914–0.931)	0.8523	−5.480 (−8.287; −2.673)	Upper: 90.07 (85.271; 94.869);
					Lower: −101.03 (−105.829; −96.231)
Once	Once	0.890 (0.878−0.902)	0.7926	−5.480 (−9.924; −1.036)	Upper: 111.893 (105.998; 117.787);
					Lower: −122.853 (−128.747; −116.958)
*Eimeria* spp. oocysts					
Once	Thrice	0.919 (0.902–0.933)	0.8446	−10.154 (−15.103; −5.205)	Upper: 86.904 (78.438; 95.369);
					Lower: −107.2118 (−115.677; −98.746)
Once	Twice	0.908 (0.898–0.918)	0.8253	−10.154 (−13.145; −7.163)	Upper: 91.645 (86.532; 96.757);
					Lower: −111.953 (−117.065; −106.840)
Once	Once	0.873 (0.858–0.886)	0.7617	−10.666 (−14.120; −7.212)	Upper: 106.905 (101.001; 112.810);
					Lower: −128.238 (−134.142; −122.333)
*Moniezia* spp.					
Once	Thrice	0.9896 (0.987–0.991)	0.9793	−1.58 (−3.360; 0.1996)	Upper: 33.319 (30.275; 36.362);
					Lower: −36.478 (−39.522; −33.434)
*Trichuris* spp.					
Once	Thrice	−0.0166 (−0.1162–0.0832)	0.0623	−0.309 (−0.544; −0.073)	Upper: 4.317 (3.914; 4.720);
					Lower: −4.934 (−5.338; −4.531)

There was no significant difference between the slopes and intercepts of the linear regression between the three comparisons (ANCOVA with Tukeyʼs multiple comparison test; *P* ​= ​1.00). However, the correlation coefficient of the comparison between Mini-FLOTAC and averaged triplicate of McMaster (*r* ​= ​0.935) was significantly higher than the comparison between Mini-FLOTAC and single replicates of McMaster (*r* ​= ​0.890) (Fisherʼs Z test; *P* ​< ​0.05). The correlation coefficient of the comparison between Mini-FLOTAC and averaged duplicate of McMaster (*r* ​= ​0.923) was also significantly higher than the comparison between Mini-FLOTAC and single replicates of McMaster (*r* ​= ​0.890) (Fisher’s Z test; *P* ​< ​0.05). There was no difference between the correlation coefficients of the comparison between Mini-FLOTAC and averaged duplicate of McMaster (*r* ​= ​0.923) and the comparison between Mini-FLOTAC and averaged triplicate of McMaster (*r* ​= ​0.935) (Fisher’s Z test; *P* ​= ​0.0679).

In the Bland-Altman analysis, differences in EPGs generated from single technical replicate McMaster had the highest number of values lying outside the limits of agreement (Fig. 3B) while the technical triplicate McMaster had the lowest number of difference values lying outside the limits of agreement (Fig. 1B). The Bland-Altman analysis for the difference values generated from averages of two replicate McMaster counts were between the values of the single and averaged triple technical replicates (Fig. 2B).

### 3.4 Comparison of Mini-FLOTAC and McMaster for *Eimeria* oocysts

**Fig. 4:**
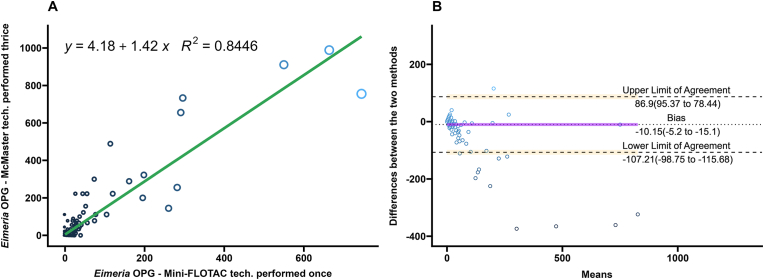
**A** Scatterplot and linear regression for *Eimeria* spp. oocysts determined by Mini-FLOTAC and three averaged technical replicates of McMaster techniques. Equations and regression coefficients are included. **B** Bland-Altman plot comparing the differences of the *Eimeria* spp. oocyst counts of the Mini-FLOTAC and three averaged technical replicates of McMaster techniques. Upper limit of agreement, lower limit of agreement, and bias are included.

**Fig. 5:**
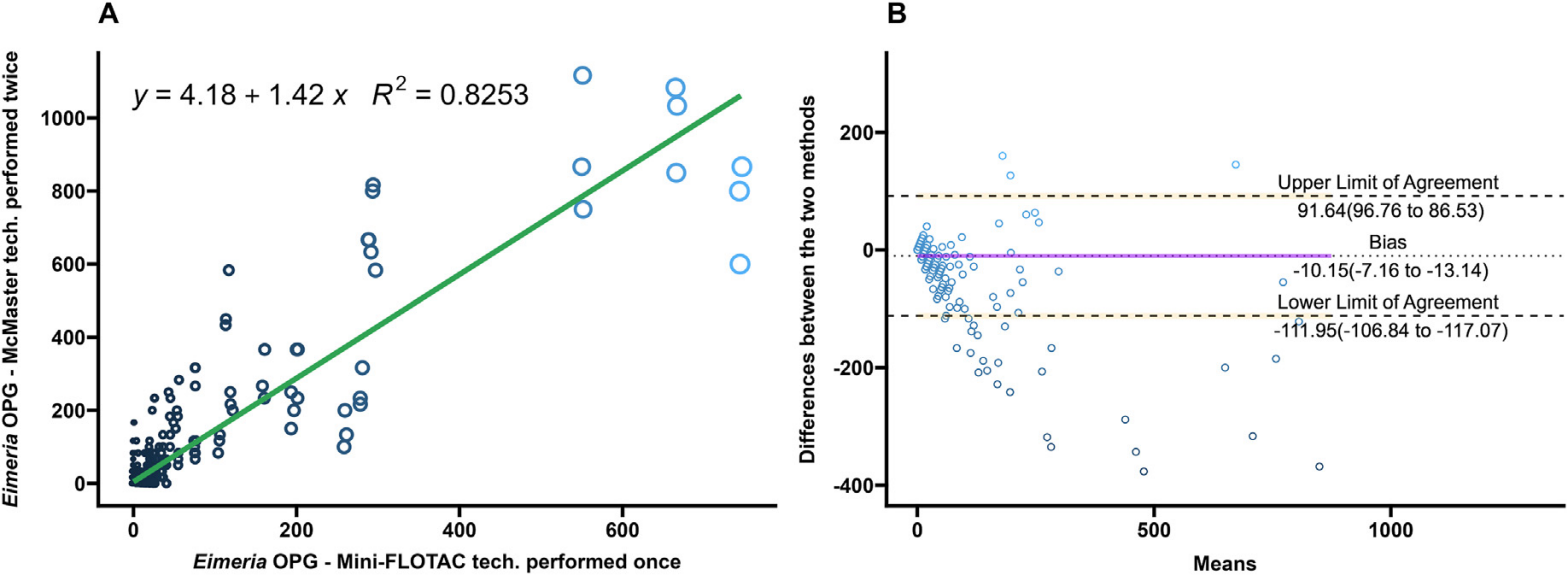
**A** Scatterplot and linear regression for *Eimeria* spp. oocysts determined by Mini-FLOTAC and two technical replicates of McMaster techniques. Equations and regression coefficients are included. **B** Bland-Altman plot comparing the differences of the *Eimeria* spp. oocyst counts of the Mini-FLOTAC and two technical replicates of McMaster techniques. Upper limit of agreement, lower limit of agreement, and bias are included.

**Fig. 6:**
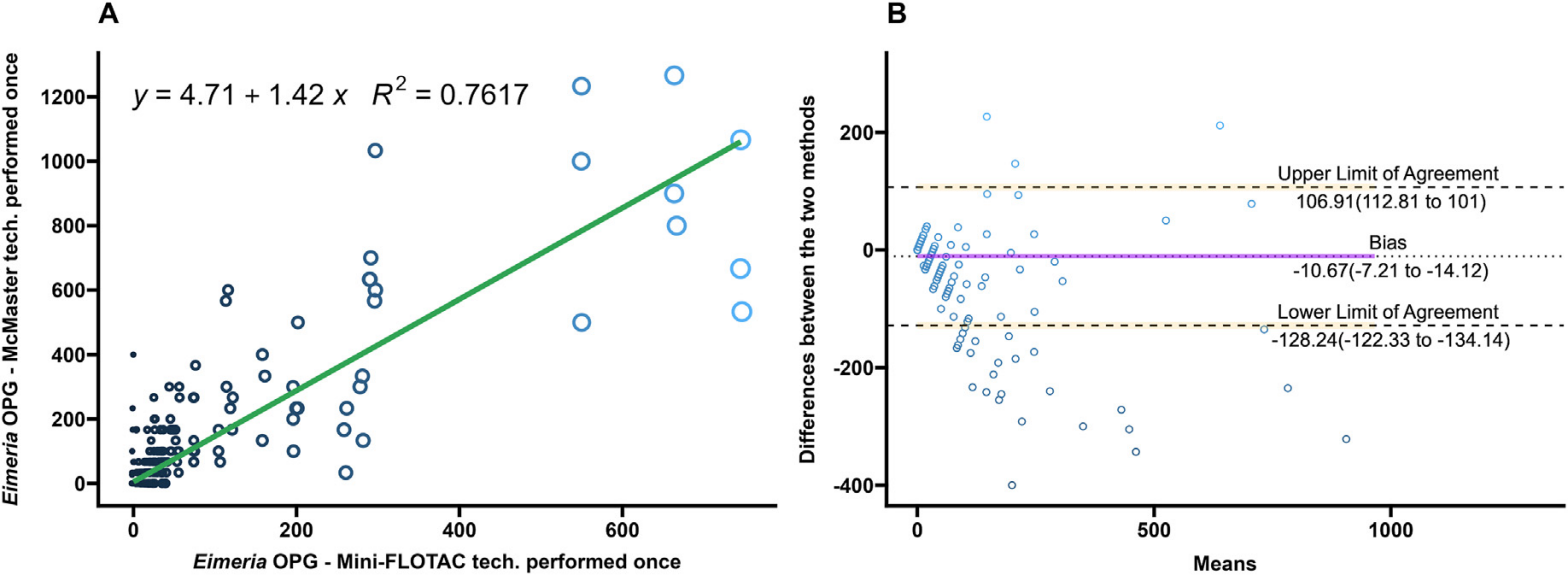
**A** Scatterplot and linear regression for *Eimeria* spp. oocysts determined by Mini-FLOTAC and one technical replicate of McMaster technique. Equations and regression coefficients are included. **B** Bland-Altman plot comparing the differences of the *Eimeria* spp. oocyst counts of the Mini-FLOTAC and one technical replicate of McMaster technique. Upper limit of agreement, lower limit of agreement, and bias are included.

A comparison of the performance of the Mini-FLOTAC and different replicates of the McMaster techniques on *Eimeria* spp. oocysts from bison fecal samples performed after the multiplication factor is summarized in Table 1. Regression and correlation coefficients were high when three technical replicates of McMaster were compared to the single replicate of Mini-FLOTAC (*R*^2^ ​= ​0.844; Pearsonʼs *r* ​= ​0.92) (Fig. 4A). Coefficients decreased when two replicates of the McMaster were averaged or only one technical replicate was performed (*R*^2^ ​= ​0.825, Pearsonʼs *r* ​= ​0.91 for two replicates; *R*^2^ ​= ​0.762, Pearsonʼs *r* ​= ​0.87 for one replicate) (Fig. 5, Fig. 6A).

There were no significant differences between the slopes and intercepts of the linear regression between the three comparisons (ANCOVA with Tukeyʼs multiple comparison test; *P* ​= ​1.00). The correlation coefficient of the comparison between Mini-FLOTAC and averaged triplicate of McMaster (*r* ​= ​0.92) was significantly higher than the comparison between Mini-FLOTAC and single replicates of McMaster (*r* ​= ​0.87) (Fisherʼs Z test; *P* ​< ​0.05). The correlation coefficient of the comparison between Mini-FLOTAC and averaged duplicate of McMaster (*r* ​= ​0.91) was also significantly higher than the comparison between Mini-FLOTAC and single replicates of McMaster (*r* ​= ​0.87) (Fisher’s Z test; *P* ​< ​0.05). There was no difference between the correlation coefficients of the comparison between Mini-FLOTAC and averaged duplicate of McMaster (*r* ​= ​0.91) and the comparison between Mini-FLOTAC and averaged triplicate of McMaster (*r* ​= ​0.92) (Fisher’s Z test; *P* ​= ​0.1292).

The Bland-Altman analysis for *Eimeria* spp. oocysts, of the singular technical replicate had the highest number of OPG values lying outside the limits of agreement. The Bland-Altman analysis of the technical duplicate had fewer OPG values lying outside the limits of agreement than the singular technical replicate. The Bland-Altman analysis of the technical triplicate had the lowest number of OPG values lying outside the limits.

### 3.5 Comparison of Mini-FLOTAC and McMaster for *Moniezia* spp. and *Trichuris* spp. eggs

**Fig. 7:**
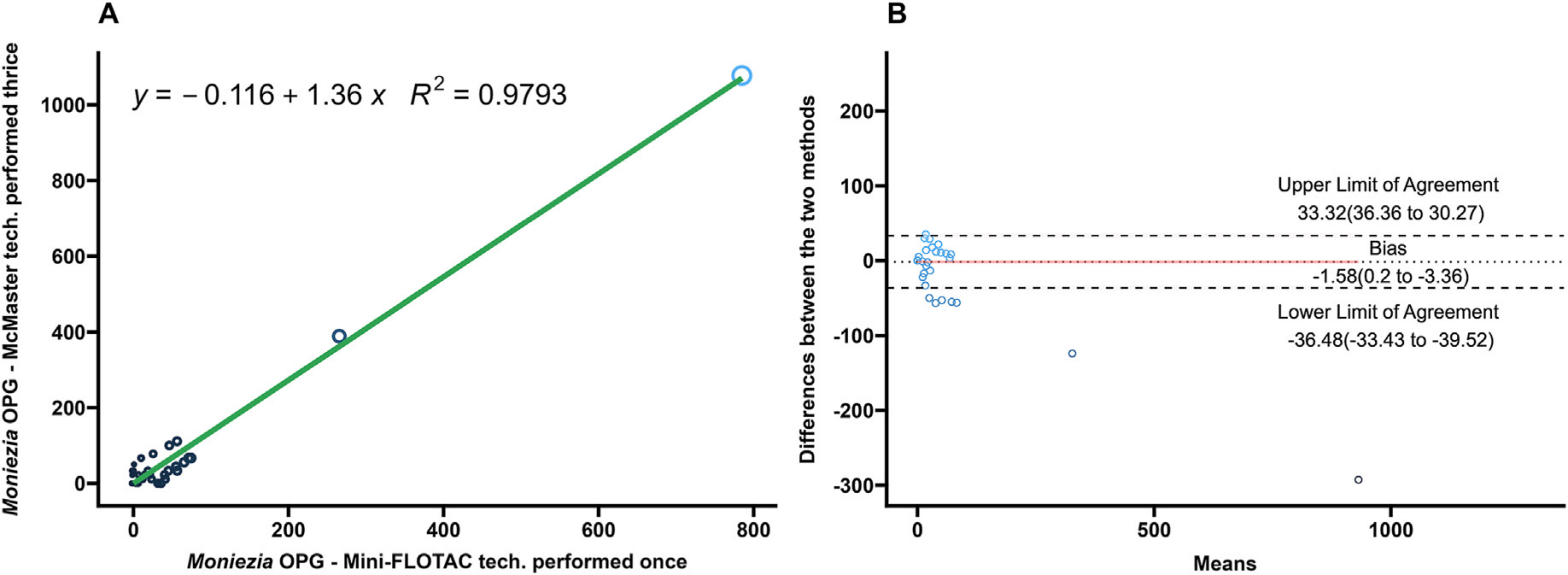
**A** Scatterplot and linear regression for *Moniezia* spp. eggs determined by Mini-FLOTAC and three averaged technical replicates of McMaster techniques. Equations and regression coefficients are included. **B** Bland-Altman plot comparing the differences of the *Moniezia* spp. egg counts of the Mini-FLOTAC and three averaged technical replicates of McMaster techniques. Upper limit of agreement, lower limit of agreement, and bias are included.

**Fig. 8:**
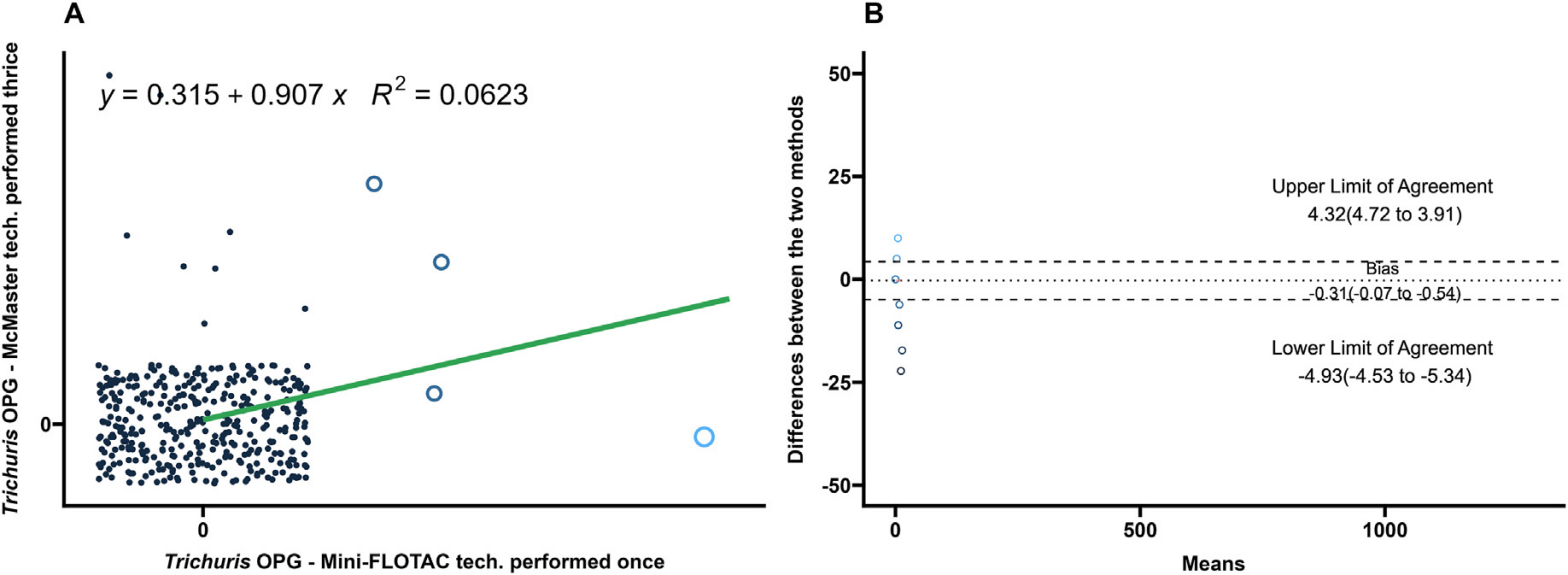
**A** Scatterplot and linear regression for *Trichuris* spp. eggs determined by Mini-FLOTAC and three averaged technical replicates of McMaster techniques. Equations and regression coefficients are included. **B** Bland-Altman plot comparing the differences of the *Trichuris* spp. egg counts of the Mini-FLOTAC and three averaged technical replicates of McMaster techniques. Upper limit of agreement, lower limit of agreement, and bias are included.

Performance of the Mini-FLOTAC and average of the triple technical replicates of the McMaster for quantification of *Moniezia* spp. and *Trichuris* spp. eggs were compared by regression, correlation, and Bland-Altman analyses and summarized in Table 1. The agreement between the two techniques was high for *Moniezia* spp. eggs (*R*^2^ ​= ​0.98; Pearsonʼs *r* ​= ​0.99) (Fig. 7A). However, the agreement between the two techniques was low for *Trichuris* spp. eggs (*R*^2^ ​= ​0.06; Pearsonʼs *r* ​= ​−0.02) (Fig. 8A). In the Bland-Altman analysis, the high numbers of zero counts resulted in narrow limits of agreement for both parasites.

### 3.6 Prevalence and overdispersion of parasites

**Fig. 9:**
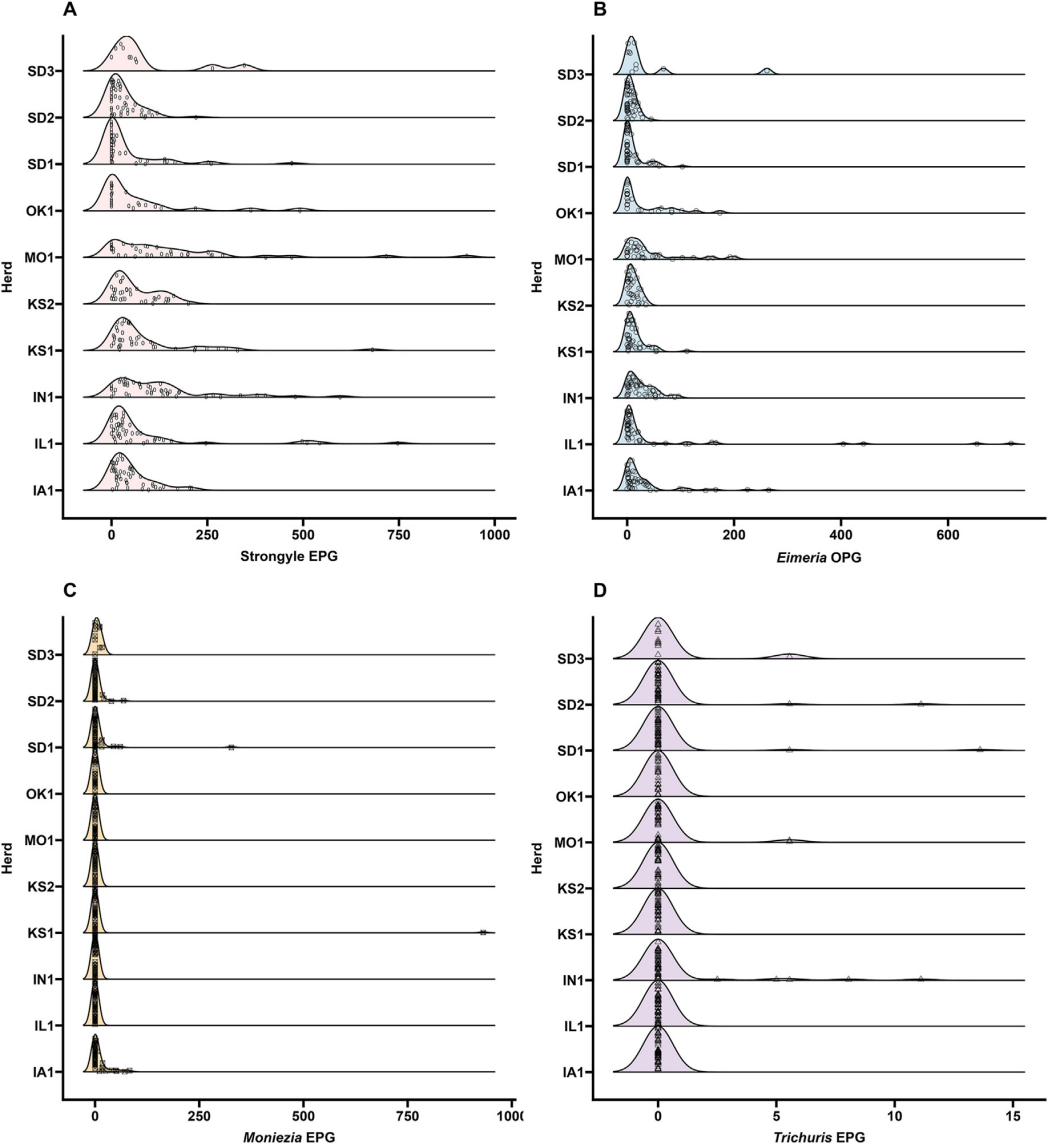
Overdispersion of gastrointestinal parasites in bison herds in this study. Ridgelines depict the distribution of averaged values from Mini-FLOTAC and triple technical replicates of McMaster counts for strongyles (EPG) (**A**), *Eimeria* spp. (OPG) (**B**), *Moniezia* spp. (EPG) (**C**) and *Trichuris* spp. (EPG) (**D**).

The overall prevalence and dispersion of strongyle eggs, *Eimeria* spp. oocysts, *Moniezia* spp. eggs, and *Trichuris* spp. eggs in each of the 10 herds was analyzed using averaged values of Mini-FLOTAC and McMaster technical triplicate counts. Prevalence was 81.4%, 73.9%, 7.5%, and 3.1% for strongyle eggs, *Eimeria* spp. oocysts, *Moniezia* spp. eggs, and *Trichuris* spp. eggs, respectively. Strongyle eggs were highly positively skewed (skewness for each herd ranged between 0.87 and 3.04) and were heavily overdispersed (Fig. 9A). *Eimeria* spp. oocyst distributions were similar to strongyle egg distributions (skewness for each herd ranged between 0.90 and 3.15) (Fig. 9B). Distributions of *Moniezia* spp. and *Trichuris* spp. were skewed when eggs were present (skewness for each herd ranged between 0.72 and 6.78 for *Moniezia* spp. 3.16 and 5.80 for *Trichuris* spp.) (Fig. 9C and D).

## 4 Discussion

In this study, the performance of two quantitative fecal techniques, a single replicate of the Mini-FLOTAC and different averaged technical replicates of the modified McMaster, were compared in 387 naturally infected North American bison in 10 herds from the Central Great Plains region of the USA. Comparative performance of the two tests varied with the parasite tested. Generally, correlation between the two techniques was higher when more technical replicates of the McMaster were used and averaged.

Several studies comparing the Mini-FLOTAC and the McMaster techniques have focused on strongyle egg counts. In ruminants, such comparisons have been performed on cattle samples (Bosco et al., 2014; Dias de Castro et al., 2017; Paras et al., 2018; Amadesi et al., 2020; Elghryani et al., 2020), sheep samples (Rinaldi et al., 2014; Kenyon et al., 2016; Bosco et al., 2018; Paras et al., 2018; Alowanou et al., 2021; Vieira et al., 2021), goat samples (Silva et al., 2020; Alowanou et al., 2021; Vieira et al., 2021) and llama samples (Paras et al., 2018). *Eimeria* spp. counts has been compared in relatively few ruminant species (Silva et al., 2013; Cruvinel et al., 2021). *Moniezia* spp. and *Trichuris* spp. counts have not been compared using the two techniques. To the best of our knowledge, this is the first study to have compared the two techniques in North American bison for the four parasites.

The McMaster technique has been known to have a lower accuracy and precision when compared to the Mini-FLOTAC technique (Noel et al., 2017). The standard Mini-FLOTAC protocol when used with the fill-FLOTAC has a sensitivity of 5 EPG (Cringoli et al., 2017). In this study, we obtained an analytical sensitivity of 5 EPG and 33.33 EPG by using the fill-FLOTAC with the Mini-FLOTAC disc and two-chambered McMaster slide, respectively. This cross-over design was shown to yield higher egg counts than traditional McMaster dilution techniques in equine strongyles (Went et al., 2018). Using the fill-FLOTAC aided in the uniform homogenization of the fecal slurry.

Both the Mini-FLOTAC and the McMaster use similar resources when used with the fill-FLOTAC. However, a single replicate of the McMaster technique can be performed relatively quicker than a single replicate of the Mini-FLOTAC (7 ​min/sample *vs* 13 ​min/sample) (Barda et al., 2014). A triplicate count of the McMaster can be performed in approximately 18 ​min (Noel et al., 2017).

Selection of diagnostic test for fecal egg count reduction is included in recent WAAVP guidelines (Geurden et al., 2022). A fecal counting technique with a sensitivity of ≤ 5 EPG is recommended for cattle (Kaplan, 2020), although no recommendations exist for bison. To obtain accurate results in FECRT, a minimum of 200 eggs in the raw counts prior to the application of the multiplication factor is recommended (Geurden et al., 2022), with a lower minimum of at least 50 eggs (Kaplan, 2020). While the Mini-FLOTAC recovered more eggs in 90.2% of samples, with fewer zero egg counts, the raw egg count threshold of 50 eggs was only reached in 6.2% of samples tested in this study. The higher recovery in the Mini-FLOTAC is explained by differences in the volume of the flotation chambers, with eggs in 2 ​ml of the fecal slurry counted in the Mini-FLOTAC technique and a volume of 0.3 ​ml in McMaster technique, and is in agreement with previous studies (Nielsen, 2021). The inability to recover 50 eggs is likely explained by overdispersion of parasites in the herds since a naturally infected population was tested and the dilution of the eggs in the large volume of feces produced by bison.

McMaster counts can be performed in triplicate with the same fecal slurry for research purposes (Slusarewicz et al., 2019), but are often performed once in diagnostic settings (Ward et al., 1997; El-Abdellati et al., 2010). In estimating composite fecal egg counts, averaging counts from greater than two McMaster slides reduced the variation due to the Poisson distribution of eggs in the fecal slurry (Morgan et al., 2005). In this study of FECs from individual bison, the differences between Mini-FLOTAC and the McMaster counts decreased as the technical replicates averaged increased from one to three. This is illustrated in the Bland-Altman plots as a decrease in the range within which 95% of the difference values were contained (limits of agreement) (Fig. 1, Fig. 2, Fig. 3, Fig. 4, Fig. 5, Fig. 6, panel B). Practically, increasing technical replicates of McMaster increased the agreement with Mini-FLOTAC values for both strongyles and *Eimeria* spp. The Mini-FLOTAC is a good alternative to the McMaster for enumeration of strongyle egg and *Eimeria* spp. oocyst counts in bison.

Due to low prevalence, the Mini-FLOTAC was compared to the average of the triplicate McMaster counts for *Moniezia* spp. and *Trichuris* spp. The two tests performed comparably for *Moniezia* spp. but not for *Trichuris* spp*.* Although *Moniezia* spp. eggs are enumerated in diagnostic laboratories routinely as part of quantitative fecal tests, the reports are not often used for therapeutic decision making by veterinarians in the field, since infections are rarely clinical. *Moniezia* spp. egg enumeration is useful in understanding drug efficacy (Chroust, 1998) and in prevalence studies (Singh et al., 2013).

As North American bison is an understudied host species, much is unknown about parasite epidemiology in this species (Huntington et al., 2019). Aggregation and overdispersion of parasites are a known phenomenon in grazing ruminants, attributed to immunocompetence of the hosts (Crofton, 1971; Barger, 1985; Sréter et al., 1994). In this study, overdispersion was evident for all four parasites studied. A few of the bison in each herd had many parasite eggs and oocysts in their feces, while the majority had few. This has implications in composite fecal egg count sampling in diagnostics (Morgan et al., 2005), in parasite transmission (Churcher et al., 2005), in effectively using chemotherapeutic intervention (Barger, 1985) and in the spread of anthelmintic resistance (Churcher & Basáñez, 2008). Aggregation and burden of parasites in bison is important to understand at both individual and herd levels since clinical sequelae is often worse in bison compared to cattle (Eljaki et al., 2016).

A prevalence of 81.4% was observed for strongyle eggs in the herds studied. This is lower than the prevalence of 100% recorded in northwest Alberta (Dies & Coupland, 2001), 100% in western South Dakota (Eljaki et al., 2016), 98.3% in western Canada (Avramenko et al., 2018), 95.2% in central Nebraska (Wiese et al., 2021) and 94% in central Canada (Woodbury et al., 2014). Since a detailed clinical history was not available for the herds in this study, lower prevalence may be explained by age, immunity, or anthelmintic treatments usage in the herds. As these strongyle eggs represent both pathogenic and relatively non-pathogenic genera, further analysis of strongyle populations at the genus and species level with the use of molecular techniques is warranted.

*Eimeria* spp. oocysts were recovered in 73.9% of the bison sampled in this study. This was in agreement with the prevalence of 73% recorded in central Nebraska (Wiese et al., 2021), 69.2% in central Canada (Woodbury et al., 2014), but lower than the prevalence of 84.5% in western Canada (Avramenko et al., 2018), and 100% reported in the western USA (Griffith et al., 2021). Of the several *Eimeria* species found in bison, *Eimeria zuernii*, *E. bovis* and *E. alabamensis* are pathogenic, while other species are non-pathogenic (Griffith et al., 2021). *Eimeria* spp. are shared in sympatric areas between bison and cattle (Ryff & Bergstrom, 1975). While identification was not performed to species level in this study, such species-level diagnostics may be warranted in herds in which *Eimeria* spp. may be a cause of significant morbidity to susceptible bison.

*Moniezia* spp. eggs were recovered in 7.5% of the bison sampled in this study. This was lower than the prevalence of 54.6% reported in northwest Alberta (Dies & Coupland, 2001), 41.6% in central Nebraska (Wiese et al., 2021), 21.9% in central Canada (Woodbury et al., 2014), and 19% in western Canada (Avramenko et al., 2018). Infection with a few tapeworms does not cause any clinical signs. However, heavily infected animals show catarrhal enteritis and intestinal hemorrhage (Demiaszkiewicz et al., 2020). Chemotherapeutic interventions are rarely warranted for *Moniezia* spp. infections.

*Trichuris* spp*.* eggs were recovered in 3.1% of the bison sampled in this study. This was lower than the prevalence of 40.9%reported in central Canada (Dies & Coupland, 2001), 30.7% in central Nebraska (Wiese et al., 2021), and 15.5% in western Canada (Avramenko et al., 2018), but higher than the prevalence of 1% reported from central Canada (Woodbury et al., 2014). Clinical disease due to *Trichuris* spp. is rare in ruminants. Acute trichuriosis may occur when young animals ingest large numbers of *Trichuris* spp. eggs in a short time period from overcrowded small indoor areas contaminated by the feces of previous cohorts (Smith & Stevenson, 1970).

Some limitations of this study were that individual bison were only sampled once, covariates such as age were not analyzed due a lack of records and the difficulty of determining age from dentition in live bison. Other covariates such as animal density on pasture, herd composition (calves, heifers, cows, bulls) and physiological status (pregnant cows, nursing cows) were not studied. Epidemiologically, these factors are known to alter the number of parasite stages shed in the samples. Accuracy and precision were not calculated for the Mini-FLOTAC and McMaster techniques since naturally infected animal samples were used, and no spiking studies were performed.

## 5 Conclusions

In conclusion, the Mini-FLOTAC is an acceptable alternative to the McMaster technique for quantitative assessment of strongyle eggs, *Eimeria* spp. oocysts, and *Moniezia* spp. eggs. The Mini-FLOTAC consistently recovered a higher number of parasites from naturally infected bison feces compared to different averaged replicates of the McMaster technique. Parasites were overdispersed in the bison and prevalence was lower than in other bison populations studied. These data add to the knowledge on the parasites of North American bison of the central USA.

## Funding

This study was funded by a grant from 10.13039/100008213the Center of Excellence for Bison Studies, South Dakota State University (JJC) and start-up funds from 10.13039/100015476Kansas State University College of Veterinary Medicine. Support for JMM during this project was provided in part by 10.13039/100005825the USDA National Institute of Food and Agriculture Hatch Project #1026173. Stipend for BWT was supported by 10.13039/100008213the Center of Excellence for Bison Studies, South Dakota State University.

## Ethical approval

Not applicable.

## CRediT author statement

Conceptualization: JRJ. Data curation: WLJ and JRJ. Formal analysis: WLJ and JRJ. Funding acquisition: JRJ. Investigation: WLJ, SR, CLA and JRJ. Methodology: JRJ. Project administration: JRJ. Resources: BW, JB, CBK, DB, JMM and JRJ. Supervision: JRJ. Validation: JRJ. Visualization: WLJ and JRJ. Writing – original draft: WLJ and JRJ. Writing - review & editing: WLJ, SR, CLA, BW, JB, JMM and JRJ.

## Data availability

The data supporting the conclusions of this article are included within the article. Raw data are available from the corresponding author upon request.

## Declaration of competing interests

The authors declare that they have no known competing financial interests or personal relationships that could have appeared to influence the work reported in this paper.

## References

[bib1] Alowanou G.G., Adenilé A.D., Akouèdegni G.C., Bossou A.C., Zinsou F.T., Akakpo G.-C.A. (2021). A comparison of Mini-FLOTAC and McMaster techniques in detecting gastrointestinal parasites in West Africa Dwarf sheep and goats and crossbreed rabbits. J. Appl. Anim. Res..

[bib2] Amadesi A., Bosco A., Rinaldi L., Cringoli G., Claerebout E., Maurelli M.P. (2020). Cattle gastrointestinal nematode egg-spiked faecal samples: High recovery rates using the Mini-FLOTAC technique. Parasit. Vectors.

[bib3] Avramenko R.W., Bras A., Redman E.M., Woodbury M.R., Wagner B., Shury T. (2018). High species diversity of trichostrongyle parasite communities within and between western Canadian commercial and conservation bison herds revealed by nemabiome metabarcoding. Parasit. Vectors.

[bib4] Barda B., Cajal P., Villagran E., Cimino R., Juarez M., Krolewiecki A. (2014). Mini-FLOTAC, Kato-Katz and McMaster: Three methods, one goal: highlights from north Argentina. Parasit. Vectors.

[bib5] Barger I.A. (1985). The statistical distribution of trichostrongylid nematodes in grazing lambs. Int. J. Parasitol..

[bib6] Bortoluzzi C., Paras K.L., Applegate T.J., Verocai G.G. (2018). Comparison between McMaster and Mini-FLOTAC methods for the enumeration of *Eimeria maxima* oocysts in poultry excreta. Vet. Parasitol..

[bib7] Bosco A., Maurelli M.P., Ianniello D., Morgoglione M.E., Amadesi A., Coles G.C. (2018). The recovery of added nematode eggs from horse and sheep faeces by three methods. BMC Vet. Res..

[bib8] Bosco A., Rinaldi L., Maurelli M.P., Musella V., Coles G.C., Cringoli G. (2014). The comparison of FLOTAC, FECPAK and McMaster techniques for nematode egg counts in cattle. Acta Parasitol..

[bib9] Chroust K. (1998). Efficacy of albendazole against *Moniezia* spp. in sheep and cattle. Acta Vet. Brno.

[bib10] Churcher T.S., Basáñez M.G. (2008). Density dependence and the spread of anthelmintic resistance. Evolution.

[bib11] Churcher T.S., Ferguson N.M., Basáñez M.G. (2005). Density dependence and overdispersion in the transmission of helminth parasites. Parasitology.

[bib12] Cringoli G., Maurelli M.P., Levecke B., Bosco A., Vercruysse J., Utzinger J., Rinaldi L. (2017). The Mini-FLOTAC technique for the diagnosis of helminth and protozoan infections in humans and animals. Nat. Protoc..

[bib13] Cringoli G., Rinaldi L., Veneziano V., Capelli G., Scala A. (2004). The influence of flotation solution, sample dilution and the choice of McMaster slide area (volume) on the reliability of the McMaster technique in estimating the faecal egg counts of gastrointestinal strongyles and *Dicrocoelium dendriticum* in sheep. Vet. Parasitol..

[bib14] Crofton H. (1971). A quantitative approach to parasitism. Parasitology.

[bib15] Cruvinel L.B., Ferreira L.L., Nicaretta J.E., Couto L.F.M., Zapa D.M.B., de Assis Cavalcante A.S. (2021). *Eimeria* spp. in naturally infected beef cattle: dynamics of oocysts excretion, prevalence, and comparison between parasitological diagnostics. Prev. Vet. Med..

[bib16] Demiaszkiewicz A.W., Pyziel A.M., Lachowicz J., Filip-Hutsch K. (2020). Occurrence of tapeworms *Moniezia benedeni* (Moniez, 1879) in European bison *Bison bonasus* L. in Białowieża Primeval Forest. Ann. Parasitol..

[bib17] Dias de Castro L.L., Abrahão C.L.H., Buzatti A., Molento M.B., Bastianetto E., Rodrigues D.S. (2017). Comparison of McMaster and Mini-FLOTAC fecal egg counting techniques in cattle and horses. Vet. Parasitol. Reg. Stud. Rep..

[bib18] Diedenhofen B., Musch J. (2015). cocor: A comprehensive solution for the statistical comparison of correlations. PLoS One.

[bib19] Dies K.H., Coupland R.W. (2001). Prevalence of gastrointestinal helminths in domestic bison herds in northwestern Alberta. Can. Vet. J..

[bib20] El-Abdellati A., Charlier J., Geldhof P., Levecke B., Demeler J., von Samson-Himmelstjerna G. (2010). The use of a simplified faecal egg count reduction test for assessing anthelmintic efficacy on Belgian and German cattle farms. Vet. Parasitol..

[bib21] Elghryani N., Crispell J., Ebrahimi R., Krivoruchko M., Lobaskin V., McOwan T. (2020). Preliminary evaluation of a novel, fully automated, Telenostic device for rapid field-diagnosis of cattle parasites. Parasitology.

[bib22] Eljaki A.A., Al Kappany Y.M., Grosz D.D., Smart A.J., Hildreth M.B. (2016). Molecular survey of trichostrongyle nematodes in a *Bison bison* herd experiencing clinical parasitism, and effects of avermectin treatment. Vet. Parasitol..

[bib23] Gałązka M., Klich D., Anusz K., Pyziel-Serafin A.M. (2022). Veterinary monitoring of gastrointestinal parasites in European bison. Int. J. Parasitol. Parasites Wildl..

[bib24] Geurden T., Smith E.R., Vercruysse J., Yazwinski T., Settje T., Nielsen M.K. (2022). World Association for the Advancement of Veterinary Parasitology (WAAVP) guideline for the evaluation of the efficacy of anthelmintics in food-producing and companion animals: General guidelines. Vet. Parasitol..

[bib25] González-Garduño R., Mendoza-de Gives P., Torres-Hernández G. (2013). Variability in the fecal egg count and the parasitic burden of hair sheep after grazing in nematode infected paddocks. Pesqui. Vet. Bras..

[bib26] Gordon H.M., Whitlock H. (1939). A new technique for counting nematode eggs in sheep faeces. J. Council Sci. Industr. Res..

[bib27] Griffith S.M., Gigley J., Fox J., Bangoura B. (2021). Identification and characterization of *Eimeria* spp. in western north American bison (*Bison bison*) herds and potential risk of cross-species transmission. Vet. Parasitol. Reg. Stud. Rep..

[bib28] Hornaday W. (1889). The extermination of the American bison.

[bib29] Huntington G., Woodbury M., Anderson V. (2019). Invited review: Growth, voluntary intake, and digestion and metabolism of North American bison. Appl. Animal Sci..

[bib30] JASP Team (2022).

[bib31] Kaplan R.M. (2020). Biology, epidemiology, diagnosis, and management of anthelmintic resistance in gastrointestinal nematodes of livestock. Vet. Clinics: Food Animal Practice.

[bib32] Kenyon F., Rinaldi L., McBean D., Pepe P., Bosco A., Melville L. (2016). Pooling sheep faecal samples for the assessment of anthelmintic drug efficacy using McMaster and Mini-FLOTAC in gastrointestinal strongyle and *Nematodirus* infection. Vet. Parasitol..

[bib33] Le Jambre L.F. (1995). Relationship of blood loss to worm numbers, biomass and egg production in *Haemonchus* infected sheep. Int. J. Parasitol..

[bib34] Levecke B., Rinaldi L., Charlier J., Maurelli M., Morgoglione M., Vercruysse J., Cringoli G. (2011). Monitoring drug efficacy against gastrointestinal nematodes when faecal egg counts are low: Do the analytic sensitivity and the formula matter?. Parasitol. Res..

[bib35] MedCalc Software Ltd (2022). https://www.medcalc.org/calc/comparison_of_proportions.php.

[bib36] Morgan E.R., Cavill L., Curry G.E., Wood R.M., Mitchell E.S. (2005). Effects of aggregation and sample size on composite faecal egg counts in sheep. Vet. Parasitol..

[bib37] Nielsen M.K. (2021). What makes a good fecal egg count technique?. Vet. Parasitol..

[bib38] Noel M., Scare J., Bellaw J., Nielsen M. (2017). Accuracy and precision of Mini-FLOTAC and McMaster techniques for determining equine strongyle egg counts. J. Equine Vet. Sci..

[bib39] Nápravníková J., Petrtýl M., Stupka R., Vadlejch J. (2019). Reliability of three common fecal egg counting techniques for detecting strongylid and ascarid infections in horses. Vet. Parasitol..

[bib40] Paras K.L., George M.M., Vidyashankar A.N., Kaplan R.M. (2018). Comparison of fecal egg counting methods in four livestock species. Vet. Parasitol..

[bib41] Rinaldi L., Levecke B., Bosco A., Ianniello D., Pepe P., Charlier J. (2014). Comparison of individual and pooled faecal samples in sheep for the assessment of gastrointestinal strongyle infection intensity and anthelmintic drug efficacy using McMaster and Mini-FLOTAC. Vet. Parasitol..

[bib42] Ryff K.L., Bergstrom R.C. (1975). Bovine coccidia in American bison. J. Wildl. Dis..

[bib44] Shamon H., Cosby O., Andersen C., Augare H., Stiffarm J., Bresnan C. (2022). The potential of bison restoration as an ecological approach to future tribal food sovereignty on the Northern Great Plains. Front. Ecol. Evol..

[bib45] Silva J.N.D., Lima M.d.L.O., Paiva R.R.L.T., Aguiar A.A.R.M., Coelho W.A.C., Pereira J.S. (2020). Comparing McMaster and Mini-FLOTAC for endoparasites diagnostic in goats. Acta Vet. Bras..

[bib46] Silva L., Vila-Viçosa M., Maurelli M., Morgoglione M., Cortes H., Cringoli G., Rinaldi L. (2013). Mini-FLOTAC for the diagnosis of *Eimeria* infection in goats: An alternative to McMaster. Small Rumin. Res..

[bib47] Singh V., Varshney P., Dash S., Lal H. (2013). Prevalence of gastrointestinal parasites in sheep and goats in and around Mathura, India. Vet. World.

[bib48] Slusarewicz M., Slusarewicz P., Nielsen M.K. (2019). The effect of counting duration on quantitative fecal egg count test performance. Vet. Parasitol. X.

[bib49] Smith H., Stevenson R. (1970). A clinical outbreak of *Trichuris discolor* infection in stabled calves. Can. Vet. J..

[bib50] Sréter T., Molnár V., Kassai T. (1994). The distribution of nematode egg counts and larval counts in grazing sheep and their implications for parasite control. Int. J. Parasitol..

[bib51] Tessaro S. (1989). Review of the diseases, parasites and miscellaneous pathological conditions of North American bison. Can. Vet. J..

[bib52] USDA-APHIS (2016).

[bib53] Vadlejch J., Petrtýl M., Zaichenko I., Cadková Z., Jankovská I., Langrová I., Moravec M. (2011). Which McMaster egg counting technique is the most reliable?. Parasitol. Res..

[bib54] Van Vuren D., Scott C.A. (1995). Internal parasites of sympatric bison, *Bison bison*, and cattle, *Bos taurus*. Can. Field Naturalist.

[bib55] Verocai G.G., Chaudhry U.N., Lejeune M. (2020). Diagnostic methods for detecting internal parasites of livestock. Vet. Clin. North. Am. Food. Anim. Pract..

[bib56] Vieira O.L., Macedo L.O., Bezerra-Santos M.A., Santos L.A.d., Mendonça C.L.d., Alves L.C. (2021). Mini-FLOTAC and McMaster egg counting method for detection of gastrointestinal parasites in small ruminants: A comparison study. Med. Vet..

[bib57] Ward M.P., Lyndal-Murphy M., Baldock F.C. (1997). Evaluation of a composite method for counting helminth eggs in cattle faeces. Vet. Parasitol..

[bib58] Went H.A., Scare J.A., Steuer A.E., Nielsen M.K. (2018). Effects of homogenizing methods on accuracy and precision of equine strongylid egg counts. Vet. Parasitol..

[bib59] Wiese J., Caven A., Zarlenga D., Topliff C., Kelling C.L., Salter J. (2021). Gastrointestinal parasites of a reintroduced semi-wild plains bison (*Bison bison bison*) herd: Examining effects of demographic variation, deworming treatments, and management strategy. Int. J. Parasitol. Parasites Wildl..

[bib60] Woodbury M.R., Copeland S., Wagner B., Fernando C., Hill J.E., Clemence C. (2012). *Toxocara vitulorum* in a bison (*Bison bison*) herd from western Canada. Can. Vet. J..

[bib61] Woodbury M.R., Lewis W.R. (2011). The efficacy of pour-on ivermectin in bison (*Bison bison*). Can. Vet. J..

[bib62] Woodbury M.R., Wagner B., Ben-Ezra E., Douma D., Wilkins W. (2014). A survey to detect *Toxocara vitulorum* and other gastrointestinal parasites in bison (*Bison bison*) herds from Manitoba and Saskatchewan. Can. Vet. J..

